# 177. AMR-Informed Antibiotic Use Metrics

**DOI:** 10.1093/ofid/ofae631.057

**Published:** 2025-01-29

**Authors:** Reid Goodman, Alice Bewley, Andrew Atkinson, Victoria J Fraser, M Cristina Vazquez Guillamet

**Affiliations:** Washington University School of Medicine, St. Louis, MO; Washington University School of Medicine in St. Louis, St. Louis, Missouri; Washington University School of Medicine, St. Louis, MO; Washington University in St. Louis, St. Louis, Missouri; Washington University School of Medicine, St. Louis, MO

## Abstract

**Background:**

Antimicrobial resistance (AMR) poses a significant public health threat, and efforts are underway to monitor and reduce unnecessary antibiotic usage. The Standardized Antibiotic Administration Ratio (SAAR) measures unit and hospital consumption of antibiotics by adjusting for facility characteristics. However, SAAR currently does not consider the prevalence rates of AMR that could justify or challenge antibiotic usage. Our objective is to develop an optimization function that identifies the best antibiotic choice for sepsis caused by Gram-negative bacilli (GNB). Optimization functions determine the optimal solution given specific constraints.

**Figure:**
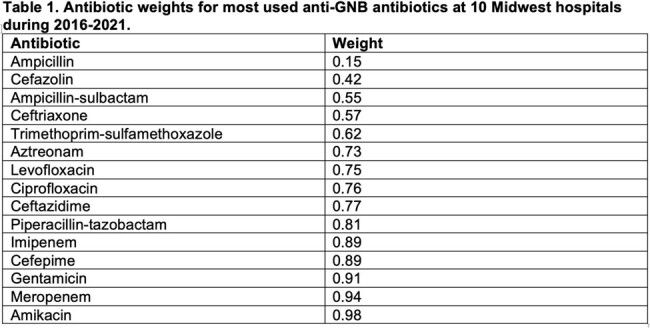

**Methods:**

This observational cohort study included all adult patients meeting the CDC sepsis criteria and admitted to any of the 10 BJC acute care hospitals between 2016 and 2021. We focused on GNB isolated from clinical cultures, specifically the 17 most common species. We selected 15 commonly used antibiotics with activity against GNB (Table 1). Initially, we calculated the antibiotic weights to determine the relative coverage of each antibiotic, expressed as the ratio of GNB susceptible to all cultured GNB. The optimization function then constrained the optimal choice to the antibiotic with the lowest weight that still exhibited activity against the ultimately isolated strain. The resulting optimal antibiotic weights were averaged per hospital and study year and reported as a ratio to the weights of antibiotics used, akin to SAAR.

**Figure:**
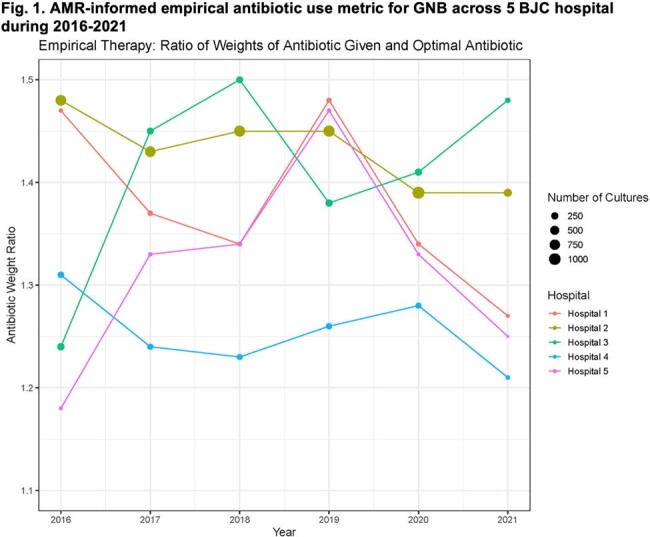

**Results:**

Our study included 11,515 GNB cultures obtained from 9,531 patients. GNB were isolated from urine (40.5%), blood (20.5%), and respiratory specimens (20.3%). We present the antibiotic weights in Table 1, along with the representative ratios of empiric and directed observed-to-optimal antibiotic weights across BJC hospitals during the study period (Figs 1 and 2). Additionally, we contrast the SAAR trends for the same hospitals in Fig. 3.

**Figure:**
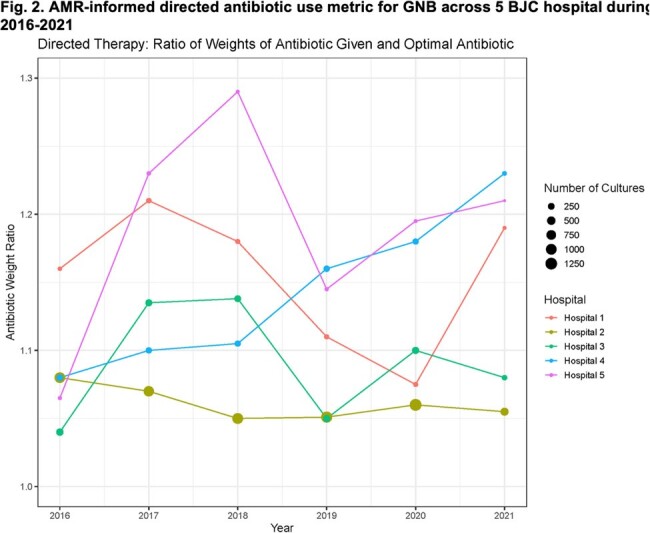

**Conclusion:**

Our optimization function selects the narrowest spectrum antibiotic and facilitates comparisons in antibiotic spectrum usage while considering existing AMR dynamics. It provides actionable insights and complements SAAR's metric of antibiotic duration.

**Figure:**
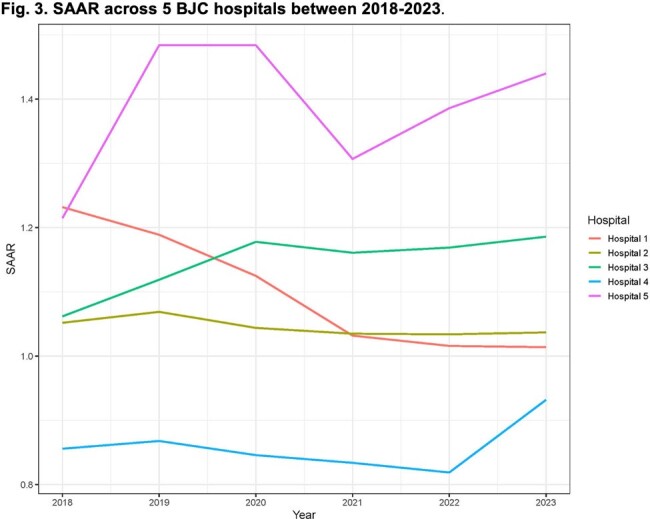

**Disclosures:**

**M Cristina Vazquez Guillamet, MD**, BNGO: Stocks/Bonds (Public Company)|Carisma Therapeutics: Stocks/Bonds (Public Company)|Ocugen: Stocks/Bonds (Public Company)

